# Integrating omics for a better understanding of Inflammatory Bowel Disease: a step towards personalized medicine

**DOI:** 10.1186/s12967-019-02174-1

**Published:** 2019-12-13

**Authors:** Manoj Kumar, Mathieu Garand, Souhaila Al Khodor

**Affiliations:** Research Department, Sidra Medicine, Doha, Qatar

**Keywords:** Crohn’s disease, Ulcerative colitis, Multi-omics, Systems biology

## Abstract

**Background:**

Inflammatory Bowel Disease (IBD) is a multifactorial chronic disease. Understanding only one aspect of IBD pathogenesis does not reflect the complex nature of IBD nor will it improve its clinical management. Therefore, it is vital to dissect the interactions between the different players in IBD pathogenesis in order to understand the biology of the disease and enhance its clinical outcomes.

**Aims:**

To provide an overview of the available omics data used to assess the potential mechanisms through which various players are contributing to IBD pathogenesis and propose a precision medicine model to fill the current knowledge gap in IBD.

**Results:**

Several studies have reported microbial dysbiosis, immune and metabolic dysregulation in IBD patients, however, this data is not sufficient to create signatures that can differentiate between the disease subtypes or between disease relapse and remission.

**Conclusions:**

We summarized the current knowledge in the application of omics in IBD patients, and we showed that the current knowledge gap in IBD hinders the improvements of clinical decision for treatment as well as the prediction of disease relapse. We propose one way to fill this gap by implementing integrative analysis of various omics datasets generated from one patient at a single time point.

## Background

Inflammatory Bowel Disease (IBD) is an inflammatory disorder of the gastrointestinal (GI) tract, resulting from the complex interactions between genetic make-up, microbiome composition, environmental factors, and mucosal immune response [1]. IBD is characterized by the repeated alternating cycles of clinical relapse and remission [2] and in the absence of an adequate treatment, a chronic inflammation leading to irreversible intestinal damages [3]. Based on the disease manifestation, IBD is classified into three major subtypes [4]: Ulcerative Colitis (UC), which primarily affects the colon, Crohn’s disease (CD) which affects various GI sites [5], and a third subtype where histology assessments done on patients do not categorize to either UC or CD. This subtype is defined as “Inflammatory Bowel Disease, type unclassified” or “Undetermined” (IBD-U) [6, 7]. IBD is a lifelong disease that substantially reduces the quality of life for the patients and their families [8].

Although the first case of UC was reported in Europe in 1875 [9] and CD was first reported in USA in 1932 [10], IBD was still a rare disease until the second half of the 20th century. Post-World War II, a rapid increase in the incidence of UC and CD had been reported, with more than 5 million people affected worldwide [11–13]. This drastic increase in IBD patterns suggest that other factors aside from industrialization must be involved in driving the changes observed in the prevalence of IBD [13, 14]. Recent studies have found a number of environmental factors including modern diet, increasing body mass index, glycemic response, medications and gut microbiota can trigger the host immune response, and have been linked to increasing IBD prevalence [15]. In addition, early childhood exposure to antibiotics, birth mode and limited childhood exposure to environmental microorganisms can also influence susceptibility to IBD development [16]. Although a great progress has been made in our understanding of IBD pathogenesis, translating this knowledge into a personalized clinical decision is still far from being achieved [17, 18]. Progress to date indicates that IBD is a multifactorial disease, therefore, a systems biology approach aiming to integrate biological omics and non-omics datasets can be a solution to resolve the complexity of the disease etiology and its heterogenous clinical outcomes. Such comprehensive approach is not only critical to provide the vast information needed for developing the best therapy or interventional strategies to IBD patients, but also for discovering clinical biomarkers that can characterize IBD pathogenesis in a subtype-specific manner. Moreover, a systems biology approach will also help with the prediction and interception of the disease and will promote personalized treatment for IBD patients.

In this review, we will summarize the results generated from various omics platforms including genomics, microbiomics, immune-proteomics, immune-transcriptomics, lipidomics and metabolomics. We will also discuss the efforts made to delineate IBD pathogenesis using these datasets and propose a framework to improve the current understanding of IBD.

## IBD etiology and diagnosis: what do we know?

**Fig. 1:**
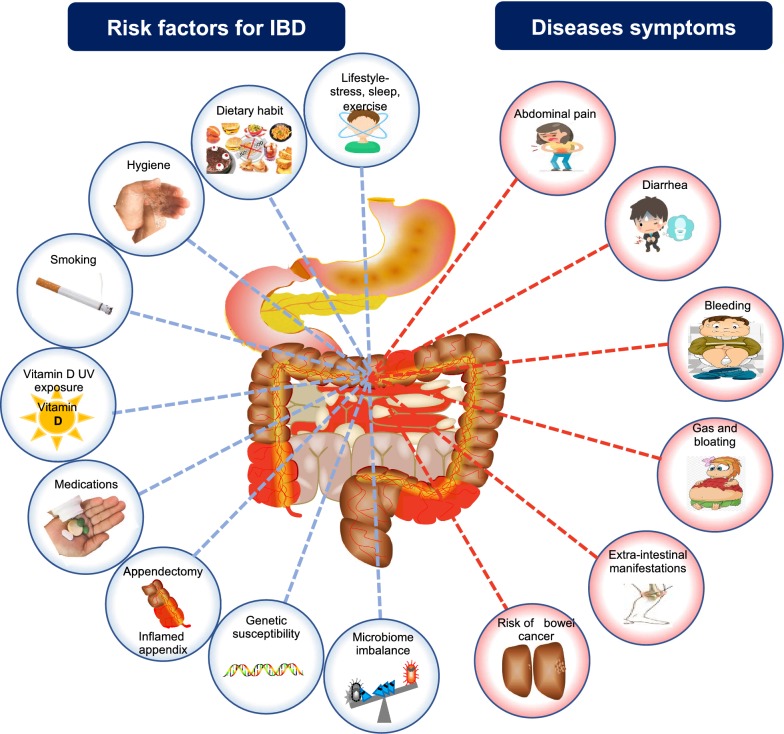
The multifaceted triggering factors for IBD and major disease symptoms. IBD develops at the intersection of host genetic predisposition, environmental influences, immune dysregulation and dysbiosis of the gut microbiota (left side). The major symptoms reported in the IBD patients are summarized on the right side

While the etiology of IBD remains exclusive, evidence indicates that the genetic make-up, mode of birth, mode of feeding at a young age, hygiene, exposure to infections, diet and stress among others are the key factors for developing IBD [15, 16]. IBD is usually suspected when the patient present with specific symptoms including diarrhea, abdominal pain, anemia and weight loss [19]. The multifaceted triggering factors of IBD and the major disease symptoms are summarized in Fig. 1. The mechanisms underlying the disease pathogenesis are not fully understood, and there is difficulty in understanding which and how-many triggering factors are involved. However, an overactive mucosal immune response and a dysbiotic gut microbiome are commonly observed in all the IBD subtypes [20, 21].

Until recently, the diagnosis of IBD seemed straightforward, as it mainly required the presence of a chronic inflammation in the GI tract with the exclusion of other causes of inflammation due to an infectious disease, vasculitides or others [22]. The current diagnostic method consists of a combination of a detailed history assessment, physical and laboratory examination, esophagogastroduodenoscopy, ileo-colonoscopy combined with histology, and imaging of the small bowel using video capsule endoscopy or enteroscopy [23–25]. Mucosal biopsies often show a characteristic appearance of UC or CD [26–28]. Small bowel imaging is recommended in all suspected cases of IBD at diagnosis; however, it can be delayed in a typical UC presentation, based on endoscopy and histology [19]. A typical UC presentation is identified by a continuous mucosal inflammation of the colon, starting from the rectum, without involving the small bowel, and with the presence of a characteristic crypt architecture disruption [4, 5, 19, 29, 30]. The inflammation is usually more severe distally and if a reverse gradient is observed, a reconsideration for the diagnosis should be prompted [4, 5, 19, 30]. It is also worth noting, that five atypical variants of UC are identified, which make the disease diagnosis and treatment more complex and often unsatisfactory [19]. The diagnosis of CD is usually based on the presence of aphthous or linear ulcers in the ileum or colon, although they can also be detected in any area of the GI tract [29]. The presence of deep serpentine ulcers along the bowel lining, and epithelioid granulomas detected in a biopsy from any area of the GI tract are sufficient to define the diagnosis of CD [4]. Endoscopy and colonoscopy are currently used for the differential diagnosis of CD and UC however, they always carry risks of bowel perforation [31].

On the other hand, the non-invasive routine laboratory investigations currently include blood testing for C-reactive protein, albumin, transaminases and erythrocyte sedimentation rate in addition to fecal testing for calprotectin and lactoferrin [30, 31]. Nonetheless, these investigations can only identify a systemic inflammation and are complementary to the invasive tests conventionally used for detecting GI tract specific flares. Serology testing can be used to subtype IBD patients: the anti-*Saccharomyces cerevisiae* antibody is found more often in CD than in UC patients and is usually associated with more severe forms of the disease. Whereas, the perinuclear antineutrophil cytoplasmic antibody is more common in UC (60–70%) as compared to CD patients [32]. However, serum positivity may be associated with other diseases which makes it harder for IBD diagnosis.

When features used to differentiate UC from CD in patients with IBD remain uncertain even after a complete workup, patients will be referred to as IBD-U until in some cases the disease develops its characteristic subtype features over time [6, 7, 33]. It is nowadays challenging to choose the best diagnostic tests and correctly classify IBD patients, especially with the increased frequency of disease heterogeneity and atypical phenotypes [19]. Moreover, clinicians are often faced with a difficult clinical decision for the IBD-U patients and frequently resort to mixing treatment protocols in an anticipation for the development of either UC or CD over time [6, 7, 33]. Such approaches often result in unsatisfactory patient outcomes, unnecessary treatment or, in some cases, inappropriate clinical care. Therefore, it is critical to understand the disease signature specific to each subtype in order to provide the most appropriate and personalized care for patients suffering from IBD.

## Application of omics: a step towards a better understanding of IBD pathogenesis

### Genomics in IBD pathogenesis

In the past two decades, technological advances in genomics and availability of large consortia genomic data have significantly contributed to our understanding of the link between specific gene loci and their relative contributions to IBD susceptibility [34, 35]. Genome-wide association studies in IBD patients have identified more than 300 genetic variants affecting various host functions—including intestinal homeostasis, epithelial barrier function, microbial composition, autophagy, production and secretion of anti-microbial peptides, and regulation of adaptive immunity [36].

Both CD and UC share around 30% of the IBD-related genetic loci [34–36], indicating that, despite being considered as two distinct IBD-subtypes with different clinical presentation, there are several common disease-related pathways such as those implicated in host immune functions, including cytokine, chemokine signaling and T helper (Th) cell responses. For example, caspase recruitment domain 9 (*CARD9*), IL-12 receptor (IL-12R), IL-23R, IL-2, IL-10, IL-21, interferon (IFN)-γ are shown to be associated with both CD and UC [36] (Table 1). On the other hand, mutation in genes such as the nucleotide-binding oligomerization domain-containing protein 2(*NOD2*) and autophagy related gene (*Atg16l1*) among others have been shown to be specific to patients with CD [37], while others like *IL1R1/IL1R2* genes are specific to patients with UC [35, 38].

**Table 1:** Some of the known gene mutations associated with IBD

Biological function	Known genetic predisposition to:		
	CD	UC	Common to CD/UC
Maintain epithelial integrity	MUC19, ITLN1	GNA12, HNF4A, CDH1, ERRFI1	
Paneth cells	NOD2, LTLN1, ATG16L1		XBP1
Innate mucosal defense	NOD2, ITLN1	SLC11A1, FCGR2A/B	CARD9, REL
IL-23/T_h_17	STAT3	IL-21	IL-23R, JAK2, TYK2, ICOSLG, TNFSF15
Restitution	STAT3	ERRFI1, HNF4A, PLA2G2A/E	REL, PTGER4, NKX2-3
Immune tolerance	IL-27, SBNO2, NOD2	IL1R1/IL1R2	IL-10, CREM
T-cell regulation	NDFIP1, TAGAP, IL-2R	IL-2, TNFRSF9, PIM3, IL-7R, TNFSF8, IFNG	TNFSF8, IL-12B, IL-23, PRDM1, ICOSLG
B-cell regulation	IL-5, IKZF1, BACH2	IL-7R, IRF5	
Solute transport	SLC9A4, SLC22A5, SLC22A4	AQP12A/B, SLC9A3, SLC26A3	
Immune cell recruitment	IL8RA/IL8RB	CCL11, CCL2, CCL7, CCL8, CCR6	MST1
Antigen presentation	ERAP2, LNPEP, DENND1B		
Autophagy	NOD2, ATG16L1, IRGM	PARK7, DAP	CUL2
Oxidative/ER stress	CAPEB4, PRDX5, BACH2, ADO, GPX1/3, SLC22A4, LRRK2, NOD2	SERINC3, HSPA6, DLD, PARK7	ORMDL3, XBP1, CARD9, UTS2, PEX13
Intracellular logistics	VAMP3, FGFR1OP, FASLG, THADA	TTLL8, CAP72, TPPP, ARPC2, LPS1, AAMP, DAP	KIF21B, PUS10, MST1
Metabolism	GCKR		SLC2A4RG

Taken together, these findings suggest that genetic predisposition plays an important role in IBD pathogenesis. However, there is still a critical knowledge gap in understanding the IBD etiology, as patients without known genetic predisposition can still develop the disease, suggesting that genomics alone is not enough to reveal the complex IBD puzzle.

### Microbiomics in IBD: from postulated theories to known differential microbial signatures

Our understanding of the human microbiome in health and disease has significantly expanded owing to the establishment of the 16S rRNA gene sequencing of the microbial genomes. The technology has propelled research on microbiome composition and function, as well as it allowed us to understand the effect of various factors in modifying the microbiome composition [39–43].

The human GI tract is densely populated by trillions of microbes including bacteria, viruses, fungi, and protozoa [39, 44]. The microbiota is continuously shaped by the exposure to a wide array of antigens found in the GI community. A healthy gut microbiota is composed of four predominant bacterial phyla, with *Firmicutes* and *Bacteroides* accounting for more than 87% of the GI microbial communities [45]. The number and composition of the microbial communities also vary in different parts of the GI tract [46, 47]. The microbiota plays an important role in maintaining the integrity of the gut epithelial barrier, food digestion, synthesis of vitamins and biomolecules, and development of mucosal immune cells among many other functions [41, 48]. In turn, the GI tract environment supports the growth, reproduction, and longevity of the gut microbial communities to maintain a state of symbiosis [49].

In the healthy state, the gut homeostasis is maintained [50]. The intestinal immune responses are regulated in order to provide a protective immunity against potential invading pathogens, while limiting any immune reaction in response to innocuous microbes and dietary antigens [50, 51]. Changes in the gut microbial compositions or microbial dysbiosis, is defined as a decrease in the intestinal microbial diversity resulting in an imbalance between commensal “protective” versus potential pathogens “harmful”, thus promoting an excessive intestinal inflammation [52]. When persistent, this response can induce a chronic, unregulated intestinal inflammation that is observed in various human diseases such as IBD, irritable bowel syndrome, asthma, obesity, cardiovascular diseases, kidney diseases, to name a few, from the wide array of diseases known to present with gut dysbiosis [39, 41, 53–72].

#### Role of the microbiome in IBD: postulated theories

**Fig. 2:**
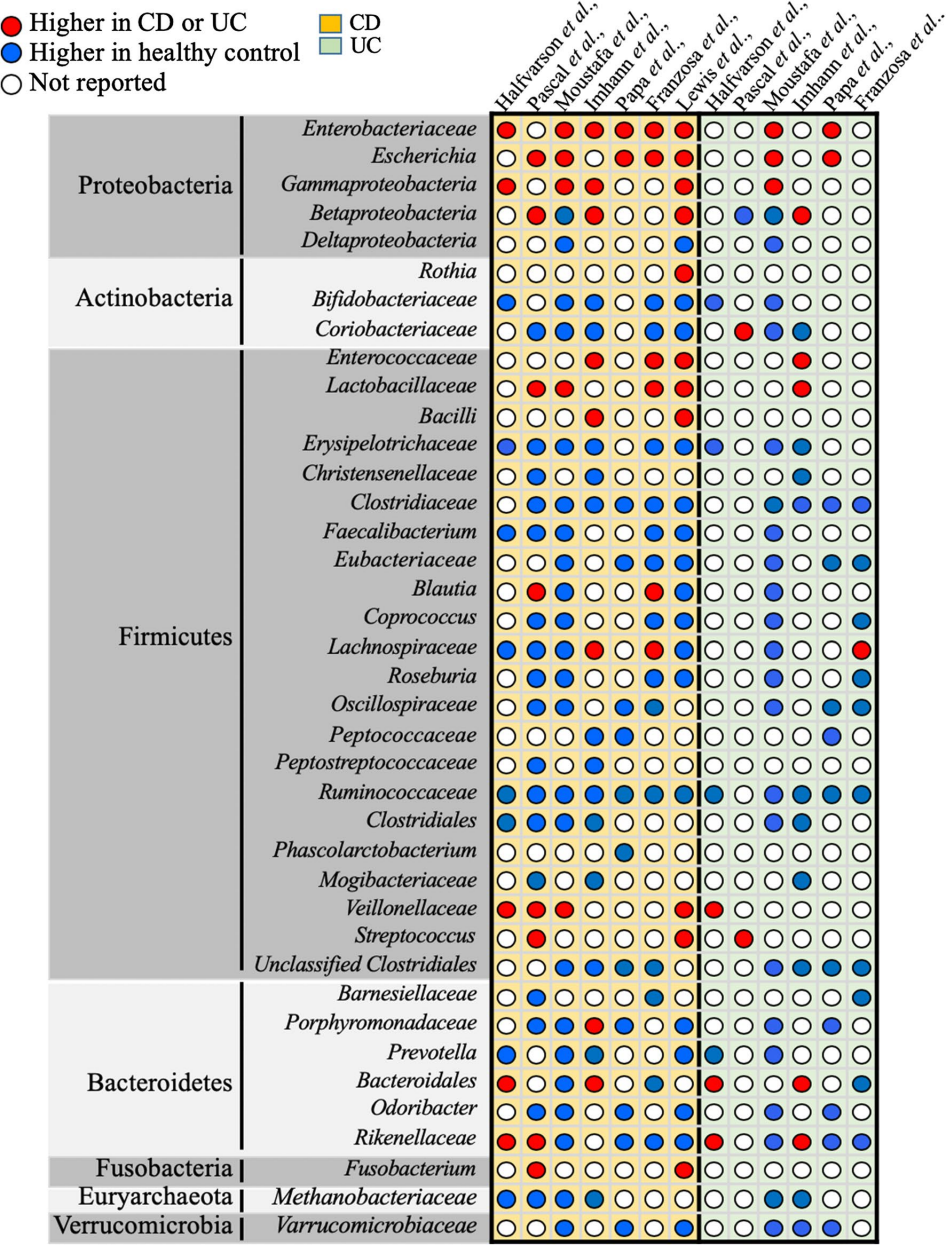
Gut microbiota dysbiosis in CD or UC patients. Qualitative comparison of relative microbial dysbiosis in CD and UC patients, retrieved from different original studies (Halfvarson et al., Pascal et al., Moustafa et al., Imhann et al., Papa et al., Franzosa et al., and Lewis et al.,). The relative increase or decrease in microbial levels is represented by red or blue dots respectively. White dots represent data not reported
B. *Presence of a potential pathogen*: IBD may be driven by a persistent pathogen (such as members of *Proteobacteria*) that contributes and exacerbates the disease pathogenesis. Members of the phylum *Proteobacteria*, specifically *Escherichia coli*, are frequently found at higher ratios in IBD patients as compared with the healthy individuals [86–88]. Strains of the adherent-invasive *E. coli* (AIEC) were isolated from the ileal mucosa in patients with CD, along with an increase in TNFα secretion [89–99]. On the other hand, AIEC is usually considered a commensal microbe present in healthy individuals, which suggests that facultative pathogens can cause disease in susceptible hosts [81, 86, 91, 100–103]. *Mycobacterium avium* subspecies, *Paratuberculosis*, and *Pseudomonas aeruginosa* have also been investigated as potential causes of CD, owing to their ability to induce chronic granulomatous enteritis and colitis in animals [104–107]. *Fusobacterium nucleatum* has been postulated to play a major role in the association between IBD and the development of colorectal cancer [108, 109]. Rather than acting as individual infectious agents, it is more likely that the interactions between the reported pathobionts (Table 2) and the rest of the GI microbiota underlie the disease pathogenesis [110].

An increasing amount of evidence supports that microbial imbalance in the GI tract influences the development and progression of IBD [61, 73–80]. Considering the key roles of the intestinal microbiota in the pathogenesis of IBD, the following theories have been postulated:

A. *Imbalance between protective versus harmful microbes*: The role of dysbiosis in IBD pathogenesis has been described in many studies assessing IBD patients. A decrease in *Firmicutes* and an expansion in *Proteobacteria* was observed in patients with CD, compared with the healthy controls in multiple studies [81, 82]. Similarly, a decrease in butyrate-producing species, such as *Faecalibacterium prausnitzii* and *Roseburia hominis*, has been reported in patients with UC or CD [83, 84]. In addition, studies comparing members of the same family (including twins), who are discordant for IBD, have postulated that dysbiosis is the primary causative agent of IBD [82, 85]. A summary of the microbial signatures known to be associated with CD or UC is summarized in Fig. 2.

**Table 2:** Presence of pathogenic bacteria (pathobionts) in CD or UC patients

Phylum	Family/species	Disease	References
Proteobacteria	*Campylobacter concisus*	CD/UC	[87]
	Invasive *Escherichia coli*	CD	[86, 89]
	*Pseudomonas* spp.	CD	[104]
	*Helicobacter* spp.	CD	[146]
	*Desulfovibrio* spp.	UC	[88]
Actinobacteria	*Atopobium parvulum*	CD	[107]
	*Collinsella* spp.	UC	[119]
	*Mycobacterium avium* spp.	CD	[106]
	*Pasteurellaceae*	CD	[106]
Firmicutes	*Clostridium difficile*	CD/UC	[117]
	*Ruminococcus gnavus*	CD/UC	[110]
	*Veillonellaceae*	CD	[119, 120]
	*Streptococcus* spp.	CD/UC	[119]
Fusobacteria	*Fucobacterium* spp.	CD	[108]
Ascomycota	*Clavispora lusitaniae*	CD	[118, 128]
	*Kluyveromyces marxianus*	CD	[118]
	*Candida albicans*	CD	[118, 129]
	*Saccharomyces cerevisiae*		[118]
	*Cyberlindnera jadinii*	CD	[124]
Bacteriophage	*Caudovirales*	CD	[130, 131]
C. *Dysregulated immune response*: Loss of the mucosal membrane integrity observed in IBD patients results in a dysregulated immune response caused by an excessive bacterial translocation combined with a continuous immune cells exposure to the microbial antigens. The integrity of the intestinal epithelial tract is established by tight cell junctions (TJ) that exist between GI epithelial cells and create a barrier against microbes [21]. The mucosal layer on the surface of the GI lining acts as the first line of defense against invading pathogens, while the epithelial cells act as the second level of surveillance [21]. The development and organization of the mucosal interface in the GI tract, are intimately linked with the gut microbiota, without which the immune system is immature and defective [111–114]. While dysbiosis promotes the growth of invasive pathogenic microbes, it also induces inflammation in the GI lining, leading to microbial translocation through the intestinal mucosal barrier to the mesenteric lymph nodes [111]. Changes in the continuity or the number of TJ strands in the intestinal epithelial cells are considered a hallmark in IBD patients [115]. An impairment in the signaling pathways responding to microbial components such as autophagy, IL23/IL17, and Paneth cells function have also been associated with IBD pathogenesis [116].

#### Microbiota signature for CD and UC diagnosis: are we there yet?

As discussed in the preceding section, the diagnosis of CD or UC patients primarily depend on endoscopy or colonoscopy; however, there are cases in which the characteristic morphology of either subtypes is absent, such as in the case of IBD-U patients [31].

The number of studies reporting gut microbial dysbiosis in IBD patients, particularly in CD patients, continues to grow exponentially and raise the possibility of using microbial signature as a diagnostic tool to distinguish between IBD subtypes [84, 117, 118]. However, inconsistent microbial profiles across studies and huge inter- and intra-individual variations, emphasize the need of longitudinal studies for a better understanding of the microbial pattern associated with IBD subtypes [19, 42]. To this end, many extensive longitudinal IBD cohorts were initiated with the aim to identify microbial signature for CD and UC. For instance, Pascal et al., used a frequent sampling every 3 months, and revealed eight microbial groups including *Fusobacterium*, *Escherichia*, *Faecalibacterium*, *Collinsella, Anaerostipes*, *Methanobrevibacter*, an unknown *Peptostreptococcaceae*, and an unknown *Christensenellaceae* that were differentially present in CD and UC patients [119, 120]. Similarly, in recent years many studies have combined cutting-edge methodologies to characterize differentially abundant gut microbial composition in CD and UC patients, but inconsistency in microbial signature across studies [16, 119–124] hinders the identification of universal microbial biomarker for disease prediction (Fig. 2). The discrepancy in microbial composition could be due to the complexity of interaction between the fluctuating gut microbiota and host features during disease course, in which the gut microbiota either influences other host functions or is being influenced by other factors- such as host genetic, diet, drugs, disease, life-style can shape the composition of gut microbiota [125]. Although, these factors and interactions are common but certainly not identical among patients.

To further establish a comprehensive insight into the host-microbial interaction and other intrinsic as well as extrinsic factors in IBD, Lloyd-Prince et al. recently provided the most detailed view of the microbiome, metabolome and host response in IBD patients [126]. Due to high inter-individual variations, which contributed to the majority of data variance, the researchers could not identify consistent microbial signature. However, researchers presented dynamic view of the complex interaction during active disease, which was well beyond the host-microbial interaction, involving multiple other downstream components including the metabolome, proteome and transcriptome [126, 127]. Despite the promising findings, the key challenges in these studies still hinder future progress for developing biomarkers. Including data reproducibility due to the lack of standard protocols for sample collection, storage, DNA extraction and sequencing methods [19]. More work is still needed to address the challenges in order to define CD and UC-specific microbial signature and to improve clinical decision making, especially in cases where a diagnosis is hard to be reached.

Apart from bacteria, the role of other microorganisms in IBD pathogenesis have been widely overlooked. These microorganisms include fungi, archaea, and viruses. Development of culture-independent methods such as sequencing of the 18S ribosomal subunit or the internal transcribed spacer region has allowed a comprehensive assessment of the mycobiome in human disease [128]. Gut mycobiome dysbiosis is observed in IBD patients and is characterized by an increase in *Basidiomycota* to *Ascomycota* ratio, a decrease in the proportion of *Saccharomyces cerevisiae* to *Malassezia sympodialis* and an increase in the abundance of *Candida albicans* (Table 2) [118]. Although, *Candida* remains asymptomatic in many IBD patients, immune-suppression and/or antibiotic treatment, which is commonly used in CD patients, are independently associated with the expansion of *C. albicans* [129].

Similarly, changes in bacteriophage composition in the gut of IBD patients have been described, particularly an increase in *Caudovirales* numbers was detected in ileal biopsy collected from CD patients [130, 131]. However, a direct contribution of the virome to IBD pathogenesis remains to be investigated.

### Immuno-proteomics in IBD pathogenesis

**Fig. 3:**
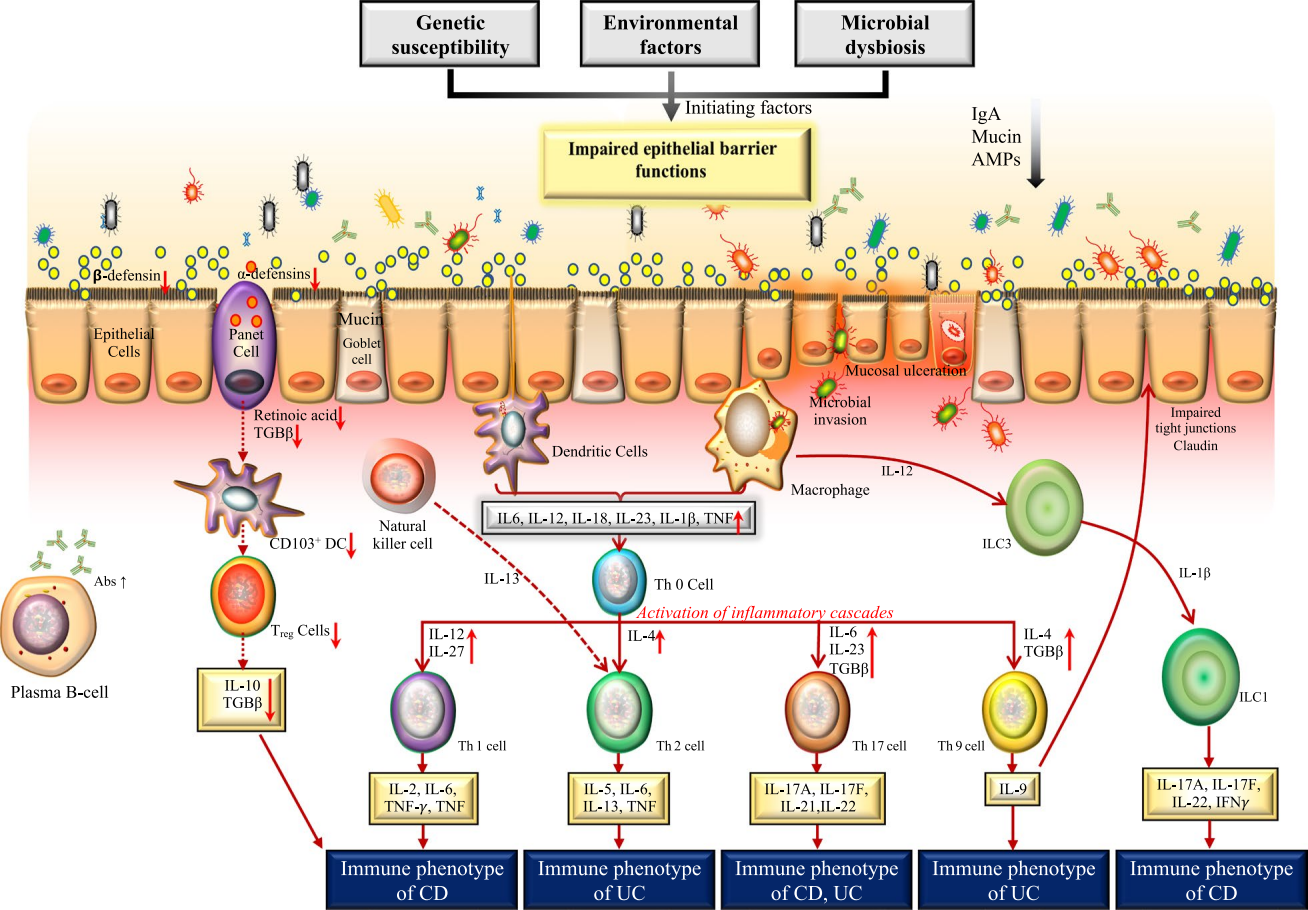
Current understanding of the Microbial–Immune interaction models in IBD. Intestinal homeostasis involves a cross-talk between the epithelial barrier functions, the immune system and the gut microbiota. The balance between pro- and anti-inflammatory cytokines in the intestinal mucosa regulates the epithelial barrier functions. In IBD, various initiating factors such as genetic susceptibility, environmental factors and microbial dysbiosis have been shown to impair the epithelial barrier functions. This results in leaky epithelial barrier resulting in microbial invasion/translocation. The translocated microbes stimulate the immune cells such as dendritic cells (DC) and macrophages leading to the activati on of an inflammatory cascade. The key cytokines produced by activated macrophages and DC (IL-12, IL-27, IL-4, 6, IL-23, TGF*b*) stimulate various T helper cell subsets (Th1, Th2, Th17, Th9) resulting in the release of cytokines that contribute to defining the immune phenotypes of CD or UC. Activated macrophages secrete IL-12 that in turn activates the innate lymphoid cell (ILC3) and ILC1 and the release of IL-17A, IL-17F, IL-22 and IFN-*γ* (yellow box). Translocated microbes result in the activation of Natural Killer T (NKT) cells. NKT-cells proliferate and differentiate into Th2 type cells via the secretion of IL-13. In homeostasis, Panet cells, located at the small intestinal crypt secrete various antimicrobial peptides (AMPs), defensins, transforming tumor necrosis factor α (TNF-α), growth factor β1 (TGF-β1) and retinoic acid. In IBD, the dysfunction of Paneth cells results in reduced AMP production and reduced signaling to regulatory T cells (Treg) resulting in a decrease of anti-inflammatory mediators. Infiltration of mucosal plasma cells is also observed in IBD patients. Black arrows indicate the direction of change in IBD. Red arrows indicate the signaling sequencing of events. *IL* interleukin, *IgA* immunoglobulin A, *AMPs* antimicrobial peptides, *DC* dendritic cells, *ILC* innate lymphoid cell, *Abs* antibodies, *TGF* transforming growth factor, *TNF* tumor necrosis factor, *IFN* interferon, *Th* T helper, *CD* Crohn’s disease, *UC* ulcerative colitis

Recent studies have highlighted a complex interplay between host genetics and environmental factors in the perturbation of the host epithelial barrier function. Thereby allowing the translocation of microbial antigens into the bowel wall, which results in aberrant immune response in the mucosal layer [132, 133]. Excessive cytokines production in the mucosal layer, not only induces intestinal inflammation and associated clinical symptoms of IBD, but also induces the systemic effects of IBD [134, 135]. For example, a reduced expression of antimicrobial peptides in the mucus layer and epithelial junction proteins like E-cadherin and claudins has been observed in IBD patients [136–138]. This in turn supports the excessive microbial growth in the mucus layer and helps the microbial translocation through the impaired epithelial barrier. The ingested microbial components can result in an overactivation of the different immune functions, leading to intestinal inflammation. CpG oligodeoxynucleotides in inflamed bowel have been shown to stimulate TLR9 signaling and induce the production of IFN by mucosal dendritic cells [116, 139]. IFNs can promote epithelial impairment or production of pro-inflammatory cytokines (Fig. 3). Following activation, macrophages residing in the lamina propria produce a large amount of IL-6, IL-18 and IL-23 into the impaired epithelium [140]. These cytokines can activate nearby antigen presenting cells (APC) and T cells (Fig. 3); where both T helper (Th)1 and Th2 cell subsets have been shown to play a crucial role in the inflamed bowel wall by secreting large amounts of pro-inflammatory cytokines [141]. Different cytokine profiles have also been observed between IBD subtypes: CD patients have a more pronounced Th1 response and produce larger amount of IL-2 and INF-gamma compared with UC patients [142]. Several interleukins are now targeted for IBD therapy [143] but with inconsistent efficacy [144], suggesting the importance of immuno-proteomic profiling in IBD patients. It is becoming clear that there is a complex network of cytokine production/activation in the inflamed bowel [145] which, in turn, is affected by its interplay with the microbial communities, host genetics, and environmental factors.

### Immuno-transcriptomics in IBD pathogenesis: how to find disease signatures?

Robust immune-transcriptomic signatures that can differentiate between healthy subjects and IBD patients or between the different IBD subtypes can serve as reliable, clinical prognostic or diagnostic biomarkers. However, such signatures are still not well-defined. The large number of genes involved in IBD pathogenesis makes the identification of a targeted, manageable list of genes for defining a signature extremely difficult. The number of potential target genes has been growing consistently; a look at the network of signaling pathways that regulates the immune responses in IBD patients clearly highlights its complexity. Immune-gene network consists of hundreds of immune cells, including their subpopulations, that are involved in the disease pathogenesis along with their highly complex gene signatures and associated functions. Therefore, the role of immune-transcriptomics can only be understood in the context of stimulations received from other factors of IBD pathogenesis [125]. The integration of genetic information, immuno-transcriptomic signature, and proteomic profiles of IBD patients, particularly on how they change during the disease remission and relapse or in response to treatment, has a great potential to uncover novel disease-specific pathways and potential biomarkers.

## Other omics: lipidomics and metabolomics in IBD pathogenesis

Previous studies have showed differences in the metabolic and lipidomic profiles of IBD patients when compared with the healthy controls [146] or when comparing CD and UC patients [147]. Agouridis et al. showed that total cholesterol and high-density lipoprotein (HDL) cholesterol levels were lower in IBD patients as compared with the healthy controls, while low density lipoprotein (LDL) cholesterol levels were higher in the IBD patients [148] Fan et al. compared the serum lipid profile of healthy controls with those of CD and UC patients [149] and identified 33 specific lipidome signatures negatively associated with CD patients and 5 lipid species significantly correlated with UC patients. Moreover, Santoru et al. reported increased levels of diacylglycerol and *n*-acylphosphatidylethanolamines in IBD patients, when compared with the healthy individuals, while urobilin, phosphatidylcholine, urobilinogen, phosphatidic acid phosphatidylserine, phosphatidylcholine and ceramide were decreased [77]. Recent analysis of the lipidome profiles of IBD patients also reflected the role of several pathways that are crucial for epithelial homeostasis, including barrier function and innate immune response [150]. Moreover, Scoville et al. showed that the serum metabolomic profile in IBD patients reflected differences in a number of lipid, amino acid, and tricarboxylic acid cycle related metabolites when compared to the healthy controls [151]. In a more recent study, Murgia et al. showed that using metabolomics and lipidomics, they were able to differentiate between IBD patients and healthy controls, and more importantly between CD and UC patients [152]. These studies illustrate the value of lipidomics and metabolomics in identifying potential biomarkers, however they remain limited and require validation in bigger cohorts.

## Conclusion

A significant progress has been achieved across multiple omics layers ranging from the genome, transcriptome, proteome, metabolome, microbiome, etc.…. Nonetheless, despite this advancement, multiple studies still assess those omics independently without taking into consideration of their complex interactions in health or disease. Hence, stemming from a single focal point, one omics approach performed at a time can only explain one aspect of any complex disease.

Multifactorial chronic diseases, such as IBD, are considered challenging as they have limited treatment options. However, they exhibit a great variety of molecular interactions involving a complex interplay between genetics, microbiome and the immune system among others [153, 154]. Classical reductionist approaches have identified key genes or pathways when trying to characterize the cause and progression of IBD and data collected from IBD patients using single omics such as microbiomics [61, 75, 79, 97, 109, 118, 155, 156] and genomics [18, 157, 158] are also available. However, they offer an incomplete overview of the complex etiology of the disease. IBD is considered a great example for systems biology approaches and multi-omics data integration, where using multi-layered analyses to combine various omics dataset is ideal to unravel the biological complexity of the disease, along with comprehensive modelling of the interactions between host factors and the microbial functional feature, in order to define personalized treatment options. While urgently needed, an integration of all omics that can be utilized on IBD patients from a systems biology perspective is still far from being achieved.

An in-depth molecular reclassification of the IBD subtypes is required and a “deep dive” assessment of the IBD pathogenesis and progression is needed in order to achieve a comprehensive analysis of the disease. Large-scale cohorts of IBD patients are needed for systems biology applications, along with a standardization of all the protocols ranging from disease index calculation methods, time points for sample collection, collection kits, sample storage, frequency of sampling, etc.…

**Fig. 4:**
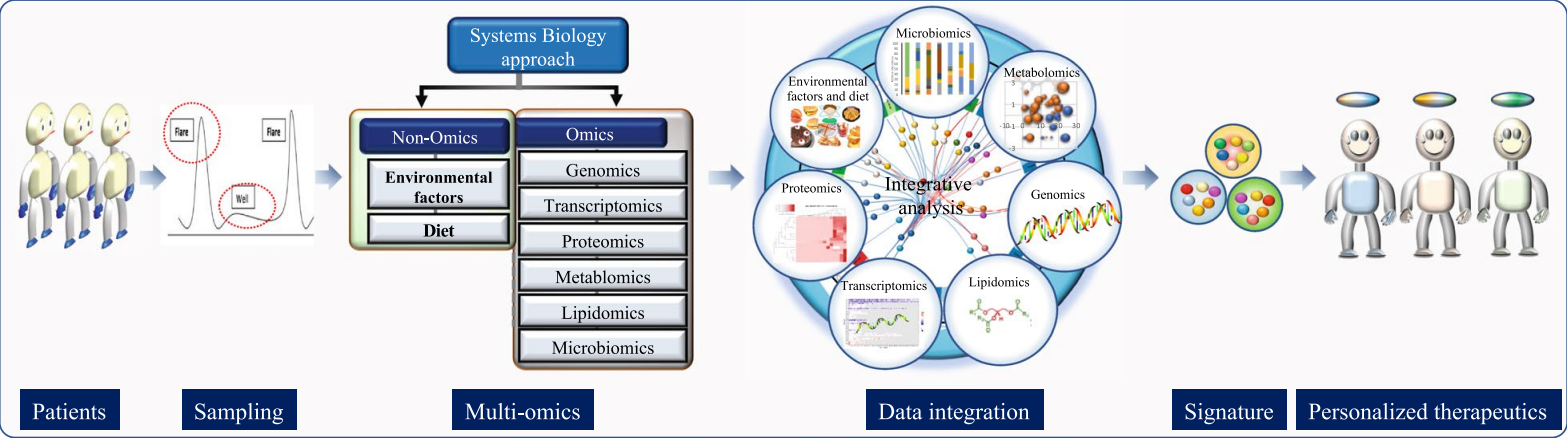
A proposed model for addressing the knowledge gap in IBD. A systems biology approach is one way to understand the complex IBD pathogenesis. Sample collection at various stages of the disease, a detailed disease index calculation, sample frequency and collection methods have to be optimized. High throughput technologies allowed processing of the samples using various omics approaches including (genomics, transcriptomics, proteomics, microbiomics, metabolomics, lipidomics and others). On the other hand, non-omics data like environmental factors, dietary information or others can also be generated. Integration of all types of omics and non-omics data will allow us to understand the phenotype-genotype interactions and help generate signatures associated with the disease. These signatures will pave the ways towards personalized treatment

In this review, we propose that integrating datasets generated at various biological layers will create a landscape, that will explain the mechanisms of interactions between those layers through the identification of key biological components from each dataset along with their functional characterization and followed by integration of each constituent using integrative analysis to identify personalized signature (Fig. 4). Moreover, this will also allow us to remove abnormal signatures across multiple molecular dimensions (Fig. 4). This unbiased and comprehensive strategy will open entirely new avenues for developing novel personalized therapeutic interventions, and help identify biomarkers that can be non-invasive, highly specific, reliable and easy to assess by clinicians in routine practices.

## Data Availability

Not applicable.

## Abbreviations

IBD: Inflammatory Bowel Disease
GI: Gastrointestinal
UC: Ulcerative colitis
CD: Crohn’s disease
IBD-U: Inflammatory Bowel Disease, type unclassified
Th: T helper cells
AIEC: Adherent-invasive *E. coli*
Tj: Tight cell junctions
